# Acquired syphilis: update on clinical, diagnostic and therapeutic aspects^⋆^^☆^

**DOI:** 10.1016/j.abd.2024.11.002

**Published:** 2025-04-10

**Authors:** Carolina Talhari, Kaique Arriel, Marcio Soares Serra, John Verrinder Veasey

**Affiliations:** a Postgraduate Program in Dermatology-Applied Sciences, Universidade do Estado do Amazonas, Manaus, AM, Brazil; b Department of Dermatology, Fundação Hospitalar Alfredo da Matta de Dermatologia, Manaus, Amazonas, Brazil; c Department of Dermatology, Universidade Santo Amaro, São Paulo, Brazil; d Department of Sexually Transmitted Infections, Sociedade Brasileira de Dermatologia Board of Directors 2023-2024, Rio de Janeiro, RJ, Brazil; e Dermatology Clinic, Hospital da Santa Casa de São Paulo, São Paulo, SP, Brazil; f Discipline of Dermatology, Faculty of Medical Sciences, Santa Casa de São Paulo, São Paulo, SP, Brazil

**Keywords:** Diagnosis, Epidemiology, Syphilis, Review, Sexually transmitted infections, Syphilis serodiagnosis, Treponema

## Abstract

Syphilis, an infection caused by *Treponema pallidum*, transmitted predominantly through sexual contact, affects several organs, causing skin, mucous membranes and systemic lesions. Despite being a secular disease, it still poses a major challenge for the public health system, since the number of cases continues to increase after years of warnings from the scientific community. Recognizing the clinical manifestations is essential for formulating the clinical hypothesis and diagnostic confirmation with complementary exams. However, recognizing skin lesions is not always simple, given the diversity of clinical manifestations which resemble other diseases. This review presents an overview of the disease, with current epidemiological data, a representation of the various clinical manifestations, a description of the pertinent diagnostic methods for laboratory confirmation, and appropriate therapeutic approaches for each clinical form.

## Introduction

Since it was first described, syphilis has been considered a stigmatizing disease. Each country whose population was affected blamed neighboring nations, or those considered enemies, for spreading the disease. Thus, syphilis has been called the French or Gallic disease, the Neapolitan disease, the Polish disease, and the Turkish disease. In the 16th century, the term “*lues venera*” (venereal plague) was used by French physician Jean Fernelius. The term “syphilis” was coined in 1530 by Italian poet and physician Girolamo Fracastoro in his work “*Syphilis, sive Morbus Gallicus*”, in which the author presents a character named Syphilus, a shepherd who, resentful of the lack of rain, blames the god Apollo for the drought that ravages his flock and afflicts his people. As punishment for his insolence, Apollo curses him with a painful and disfiguring disease that ends up spreading to the entire population, the disease of Syphilus.^1^, ^2^

Syphilis is a chronic infectious disease caused by *Treponema pallidum*, which is transmitted predominantly through sexual contact and can exhibit cutaneous and systemic manifestations. Congenital transmission occurs via the transplacental or hematogenous routes and, less frequently, transmission can also occur through blood transfusions, sharing of needles, or accidental inoculation. Humans are the only known reservoir.^3^

It is characterized by long latency periods and the ability to reach multiple organs, causing cutaneous, mucous, cardiovascular, and neurological lesions. In most cases, syphilis begins with an ulcerative lesion in the anogenital region. As with all sexually transmitted diseases, this ulceration is very important in the transmission of the human immunodeficiency virus (HIV) and hepatitis B and C. This situation is aggravated because syphilitic genital ulcers are densely infiltrated with lymphocytes (the main target cells for HIV infection) and, therefore, provide an important gateway for the acquisition of this virus.^3^

When it affects pregnant women, if left untreated, it can result in miscarriage, prematurity, neonatal death, or late manifestations of the conceptus, such as deafness, developmental deficit, and bone malformations, characterizing congenital syphilis.

There are three main theories to explain the origin of syphilis. The first, called the “pre-Columbian theory”, states that “*pinta*” was the first treponematosis to appear in the Afro-Asian region, approximately in the year 15,000 BCE, with an animal as a reservoir. Mutations in the treponema are believed to have given rise to “yaws” in 10,000 BCE, “endemic syphilis” in 7,000 BCE, and “sexually transmitted syphilis” in 3,000 BCE in Southwestern Asia, from where it spread to Europe and the rest of the world.^1^, ^2^

The “unitary theory,” considered by some authors to be a variation of the pre-Columbian hypothesis, argues that treponematoses have always had a global distribution. According to this theory, both syphilis and non-sexually transmitted treponematoses are geographic variants of the same original infection. The different clinical manifestations would be justified by adaptive responses of the treponema to the environment, cultural differences, and the miscegenation of people.^1^, ^2^

Finally, the “Colombian theory”, a very popular hypothesis accepted by many authors, states that the disease emerged in the Americas and was brought to Europe by the navigators of Christopher Columbus' fleet in 1493. This theory is supported mainly by the finding of skeletal lesions characteristic of the diagnosis of syphilis in multiple fossils, thousands of years old, found in several regions of the Americas. In Europe, the findings compatible with the presence of syphilitic alterations in human fossils are controversial and inconclusive.^1^, ^2^

## Epidemiology

In Brazil, acquired syphilis mainly affects adolescents and young adults between the ages of 15 and 25, but it can occur in any age group. The disease has no racial, gender, or socioeconomic class predilection; it is mainly associated with risky sexual behavior. It does not confer immunity, and reinfection and superinfection can occur.^3^

According to the Sexually Transmitted Diseases Bulletin of the Centers for Disease Control and Prevention (CDC) of the United States, the incidence of primary and secondary syphilis has been increasing since 2001. In 2022, 59,016 cases of primary and secondary syphilis were reported in the United States (17.7 cases per 100,000 inhabitants). From 2021 to 2022, the national rate of primary and secondary syphilis among women increased by 19.2%, with increases observed in 36 states and the District of Columbia. The increase in the number of cases among women is simultaneous to that observed among men who have sex only with women, reflecting the expanding epidemic of heterosexual syphilis in the United States. It should also be noted that men who have sex with men (MSM) account for almost half (45.1%) of all cases of primary and secondary syphilis in men in 2022.^4^

In Brazil, it became mandatory for acquired syphilis to be reported nationwide in 2010. In the period from 2012 to June 2023, 1,340,090 cases of acquired syphilis were reported in the Notifiable Diseases Information System (Sinan, *Sistema de Informação de Agravos de Notificação*). Of the total number of cases: 50.0% occurred in the southeastern region; 22.3% in the southern region; 14.2% in the northeastern region; 7.2% in the midwestern region and 6.3% in the northern region.^3^

In 2022, 213,129 cases were reported in Brazil, of which 101,909 (47.8%) were reported in the southeastern region, 46,291 (21.7%) in the southern region, 32,084 (15.0%) in the northeastern region, 16,327 (7.7%) in the northern region and 16,518 (7.8%) in the midwestern region.^3^

**Fig. 1:**
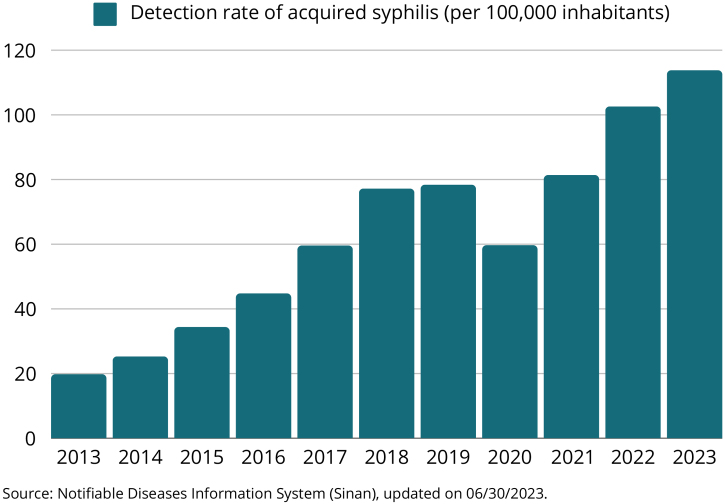
Longitudinal series of acquired syphilis incidence in Brazil since 2013, showing a progressive increase in case notifications with the exception of 2020, where there was an impact due to the COVID-19 pandemic.

Between 2012 and 2018, the acquired syphilis detection rate showed an average annual growth of 35.4%. However, in 2019, this rate remained stable and declined by 23.4% in 2020, due to the COVID-2019 pandemic. From 2021 onwards, the detection rate increased again, to a level higher than the pre-pandemic period across the country, with an increase of 23.0% in the last year (Fig. 1). Between 2021 and 2022, the growth in the rate was 26.6% (from 76.3 to 96.6 cases per 100,000 inhabitants) in the midwestern region; 24.9% (from 90.4 to 112.9 cases per 100,000 inhabitants) in the southeastern region; 24.1% (from 121.8 to 151.2 cases per 100,000 inhabitants) in the southern region; 19.1% (from 72.5 to 86.3 cases per 100,000 inhabitants) in the northern region and 15.9% (from 47.8 to 55.4 cases per 100,000 inhabitants) in the northeastern region.^3^

In 2022, 61.3% of the total cases occurred in men and the detection rates reached 234.5 and 142.5 cases per 100,000 inhabitants in the age groups of 20 to 29 years and 30 to 39 years, respectively. The number of syphilis cases in female adolescents was higher than in male individuals, representing a M:F ratio of 0.7 (seven men with syphilis for every ten women with syphilis) in 2022. On the other hand, in that same year, in the age groups of 20 to 29 years and 30 to 39 years, the M:F ratio was 1.8 (18 men with syphilis for every ten women) and 2.0 (20 men for every 10 women with syphilis), respectively.^3^

In the last 20 years, there has been a significant increase in the number of syphilis cases worldwide. This increase can be attributed to several factors, such as changes in sexual behavior and decreased fear of becoming infected with HIV. During this period, some studies identified an increase in the incidence of syphilis and other sexually transmitted infections (STIs) in individuals using HIV Pre-Exposure Prophylaxis (PREP).^5^, ^6^

In patients living with HIV (PLHIV), the prevalence of syphilis is significantly higher than in the general population. In a Chinese study, the HIV/syphilis coinfection rate was 20%. Other studies carried out in Turkey and Brazil showed rates of 23% and 11%, respectively. In the subgroup analysis, the three studies showed a significantly increased prevalence of coinfection in the MSM group, when compared to the heterosexual group. In all three studies, the majority of patients had latent syphilis.^7^, ^8^, ^9^

## Etiopathogenesis

*Treponema pallidum*, the etiological agent of syphilis, is a gram-negative, facultative anaerobic, and catalase-negative bacterium of the spirochete group. The treponema penetrates the host through small fissures in the skin or mucosa produced by sexual activity. Once inside the epithelium, it multiplies locally and invades the lymphatic vessels and bloodstream. During this invasion process, this extracellular bacterium avoids recognition and adaptation of the host's innate and adaptive immune responses due to the low expression of proteins in the plasma membrane, as well as the absence of lipopolysaccharides (highly pro-inflammatory glycolipids found in Gram-negative bacteria) and expression of lipoproteins capable of activating macrophages and dendritic cells.^10^, ^11^

Although the scarcity of PAMPs (Pathogen-Associated Molecular Patterns, molecules recognized by the innate immune system as a sign of invasion by a group of pathogenic agents) in the outer membrane of *T. pallidum* allows the bacterium to replicate locally and disseminate, the detection of pathogens by the host is eventually triggered. The treponema is captured by dendritic cells that migrate to the lymph nodes and have treponemal antigens to B and T lymphocytes. Antibodies are produced that favor the degradation of spirochetes by phagocytes, releasing lipopeptides (cardiolipins) and other PAMPs that activate T cells, ending the cascade of cytokine production such as IFN-γ, tumor necrosis factor (TNF), and IL-6.^10^, ^11^

## Clinical aspects

An old Latin saying “*Omnis syphiliticus mendax (est)”* (“Every syphilitic is a liar”) is relevant even today: one cannot be certain that the case history reported by the patient with syphilis is in accordance with the facts, especially with regard to sexual history.^12^, ^13^, ^14^ Thus, recognizing the clinical manifestations is essential for developing the clinical hypothesis and confirming the diagnosis with complementary exams. However, recognizing syphilis lesions is not always easy, since the disease is considered by most researchers to be a great imitator, given the diversity of clinical manifestations that resemble other diseases.^15^, ^16^

The association between syphilis and HIV is well established, with syphilis increasing the risk of HIV transmission. HIV infection, in turn, can alter the natural history of syphilis, making it difficult to diagnose the disease caused by *T. pallidum*.^17^, ^18^ Along the descriptions of the clinical forms, the particularities of the patient who has co-infection with these two STIs will be highlighted.

**Fig. 2:**
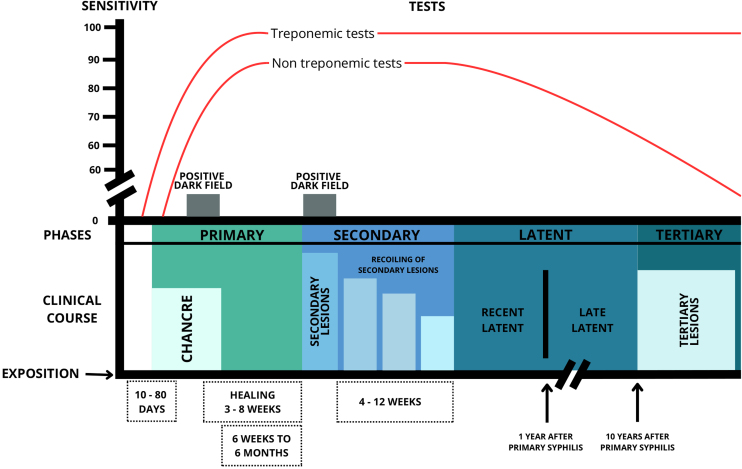
Schematic diagram of the clinical and laboratory course of untreated acquired syphilis. Adapted from: Clinical Protocol and Therapeutical Guidelines for Comprehensive Care for People with Sexually Transmitted Infections (STIs), Ministry of Health, Brazil, 2021.

Syphilis can be classified into several forms, either by the time of disease progression (early, up to one year after infection, and late, after one year) or by stages of evolution (primary, secondary, latent and tertiary).^19^ To facilitate the characterization of clinical aspects, the classification by stages of evolution was chosen in this paper (Fig. 2).

### Primary syphilis

**Fig. 3:**
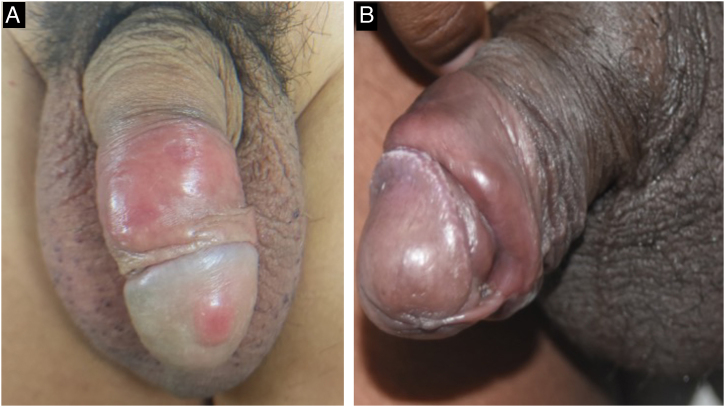
Primary syphilis lesions on the penis. A- Patient with a hard chancre lesion on the glans and two on the foreskin. B- Note the presence of a “cord-like” lesion in the balanopreputial sulcus (which can also be seen in patient A).

The classic lesion of primary syphilis is a painless chancre, which identifies the site of inoculation of the bacteria in the body. It occurs between three and 90 days after inoculation (mean 21 days) and develops from a macule to a papule and nodule, which loses its covering epithelium and then becomes an erosion. The loss of deeper tissue produces an ulcer, typically measuring 0.5 to 3 cm in diameter. The central surface of the chancre is clean, smooth, and mucoid and produces a slight serous exudate. The border is usually flat and well-demarcated. Chancres are hardened to the touch due to the surrounding edema and lymphocytic infiltration, giving rise to the name “hard chancre” for this lesion which is usually solitary. The occurrence of two or more lesions may be related to coinfection with the HIV virus (Fig. 3). It is important to note that protosyphiloma can often go unnoticed by the patient due to the painless nature of the lesion.^18^, ^20^, ^21^, ^22^, ^23^, ^24^

When the chancre is located in an area with excess skin, such as the foreskin, the presence of the “flag sign” or “button sign”, known in English as the “dory flop sign”, is evidenced during a dynamic inspection, which depicts this infiltrate and solid lesion moving *en masse*.^22^, ^24^, ^25^

Chancre redux is an uncommon form of recurrent syphilis, in which a primary syphilis lesion, usually a hard chancre, reappears at the site of the original infection after the person has been inadequately treated or has not completed treatment for the disease. This recurrence occurs due to the persistence of the bacterium *Treponema pallidum* in the body. Chancre redux is a sign that the infection has not been completely eradicated and that treatment should be reassessed to ensure the complete elimination of syphilis. It is currently a less common manifestation since treatments with appropriate antibiotics are usually effective. It should not be confused with *pseudochancre redux*, a clinical manifestation of tertiary syphilis that presents a gummatous lesion at the site of the original chancre.^22^, ^26^

Primary infection in the glans penis may present with multiple erosions of treponematous origin characterized by flat, whitish elevations that are easily confused with fungal or irritative etiology. This atypical presentation is named after its first descriptor, “Follmann’s syphilitic balanitis”, and may develop before or after the appearance of the primary chancre.^15^, ^21^, ^24^, ^27^, ^28^, ^29^

Another manifestation that has been observed at this stage is the presence of painless, hardened “cord-like” lesions on palpation, located mainly in the balanopreputial sulcus (Fig. 3).^15^

**Fig. 4:**
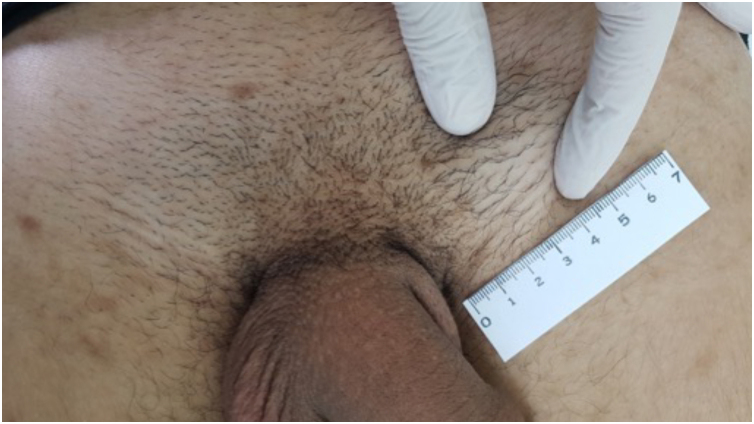
Patient with primary syphilis showing left inguinal lymph node enlargement.

The inguinal lymphadenopathy seen at this stage has been highlighted by secular researchers. It follows the lymphatic drainage chain, preferably unilateral and inflammatory, but may be bilateral: lesions on the penis and vaginal labia tend to show inguinal lymphadenopathy; anal lesions, lymphadenopathy in the pelvic and abdominal cavity; oral and labial lesions, submandibular and cervical lymphadenopathy. The presence of regional lymph node enlargement is so frequent that Fournier stated that this manifestation “follows the chancre as the shadow follows the body”, and it is also recognized as “mayor lymph node” (Fig. 4).^21^, ^24^

Co-infection of *Treponema pallidum* and *Haemophilus ducreyi*, in the same ulcerated lesion, constitutes Rollet's mixed chancre (hard chancre associated with soft chancre). This situation is rarely described in the literature; perhaps not because of the rarity of the condition, but because of the syndromic approach to genital ulcers, with a low frequency of microbial investigation for both agents in the lesion.^30^

The primary form of syphilis can last between two and eight weeks and tends to disappear spontaneously, regardless of treatment, usually without leaving a scar.^21^, ^22^, ^24^, ^31^ The chancre is identified in about 15% of patients at the beginning of the secondary stage, and cases that exhibit this concomitant primary and secondary phase should be investigated for immunosuppression as co-infection with the HIV virus.^20^, ^21^, ^32^

### Secondary syphilis

In untreated individuals, treponemas proliferate in the chancre and migrate via the lymphatic system to the bloodstream, from where they spread throughout the body. Signs and symptoms appear on average between six weeks and six months after infection and last on average between four and 12 weeks.^19^, ^22^, ^31^

Rarely, primary manifestations may be absent during the course of the infection, with the first signs of the disease represented by lesions in the secondary phase. This situation is known as “decapitated syphilis”, due to the absence of the primary phase during the course of the disease, or “*syphilis d'emblée*”, a French word that refers to the immediacy of the infection. It occurs when the treponema is inoculated directly into the bloodstream, such as untested blood transfusions or the sharing of needles.^33^

Thus, while primary syphilis represents the site of inoculation of the bacteria in the epithelium, secondary syphilis corresponds to the spread of the parasite throughout the host body. The most frequent clinical manifestations are mucocutaneous lesions (90%‒97%), with or without systemic signs and symptoms, such as generalized lymphadenopathy (50%‒85%), malaise (13%‒20%), sore throat (15%‒30%), body aches (6%‒8%), and low-grade fever (5%‒8%).^22^, ^26^, ^31^, ^34^ Involvement of internal organs such as the lungs, stomach, and intestine has been described, causing a variety of symptoms and clinical presentations.^35^, ^36^

**Fig. 5:**
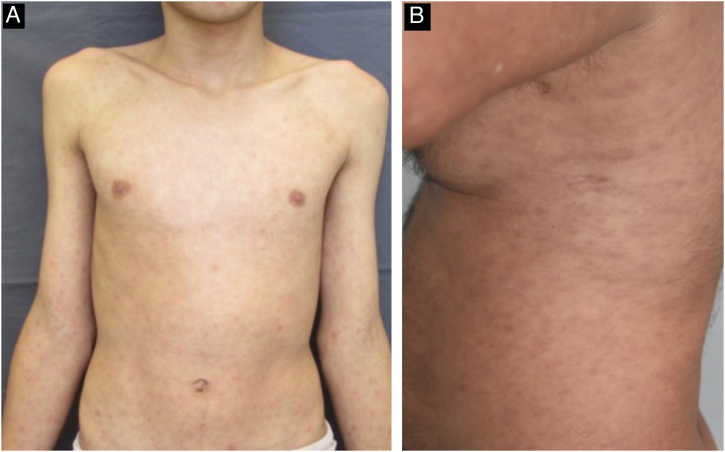
Patients with secondary syphilis; A- Patient presenting with syphilitic roseola; B- Patient with maculopapular exanthema on the trunk.

The cutaneous and mucous lesions of secondary syphilis are called syphilides, and the first cutaneous sign of this stage is a macular rash (“syphilitic roseola”) that is ephemeral, lasting a few days, with a pale erythema (“cupric erythema” or “sad erythema”) and a preference for the trunk and limbs. In melanodermic individuals, the roseola phase may go unnoticed, due to the difficulty in perceiving mild erythema on dark skin (Fig. 5).^22^, ^26^, ^31^ Residual hypochromic macules (leukoderma) may follow the regression of the roseola stage and are more common in women with dark hair. Because it typically affects the neck and shoulders, it is known as the “necklace of Venus” and may be misdiagnosed as vitiligo.^26^, ^34^

**Fig. 6:**
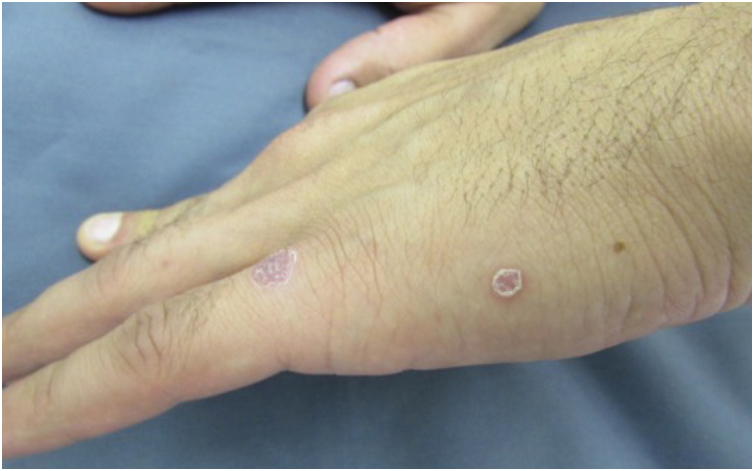
Detail of a lesion in a patient with secondary syphilis, showing Biet's collarette.

**Fig. 7:**
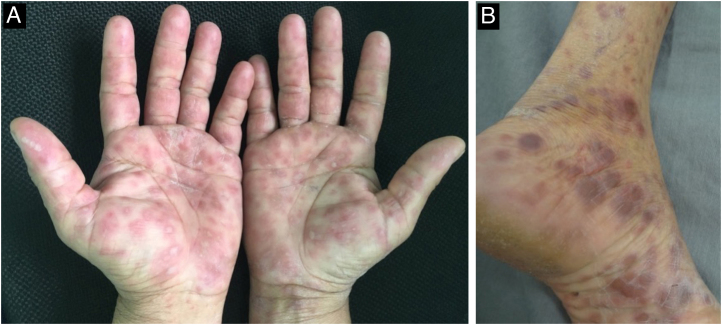
Palmoplantar involvement of secondary syphilis. A- Erythematous papules and macules on the palmar region; B- Erythematous-violaceous papular lesions on the plantar region.

The initial macular stage develops into a symmetrical papular eruption including the palms and soles, usually desquamative, with peripheral desquamation called “Biett collarette” (Fig. 6), which may also be smooth, follicular or, rarely, pustular. In this phase, the erythema is more intense and evident (Fig. 5). Vesicles do not usually occur, although vesicopustular lesions are seen on rare occasions and are common on the palms and soles (Fig. 7).^22^, ^26^, ^31^ Despite the extent of the lesions, pruritus is not a characteristic symptom of the disease. However, recent studies indicate that these eruptive lesions may be associated with this symptom, prominent eosinophilic infiltration on histopathology.^37^, ^38^

**Fig. 8:**
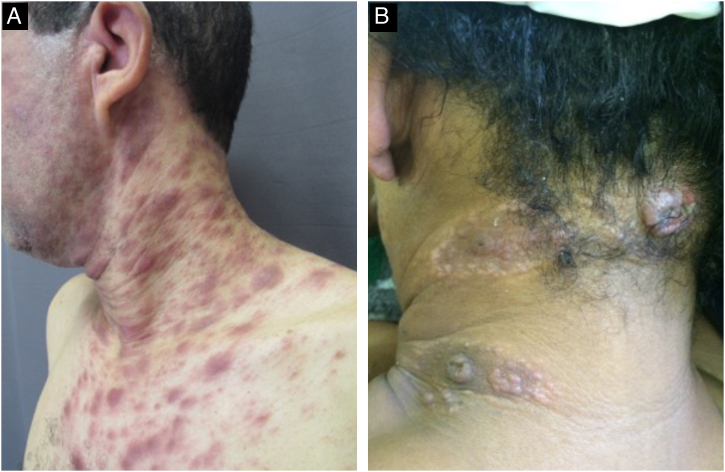
A and B-Patients with secondary syphilis showing nodular lesions in the cervical region; B- Lesions in corymbiform arrangement.

**Fig. 9:**
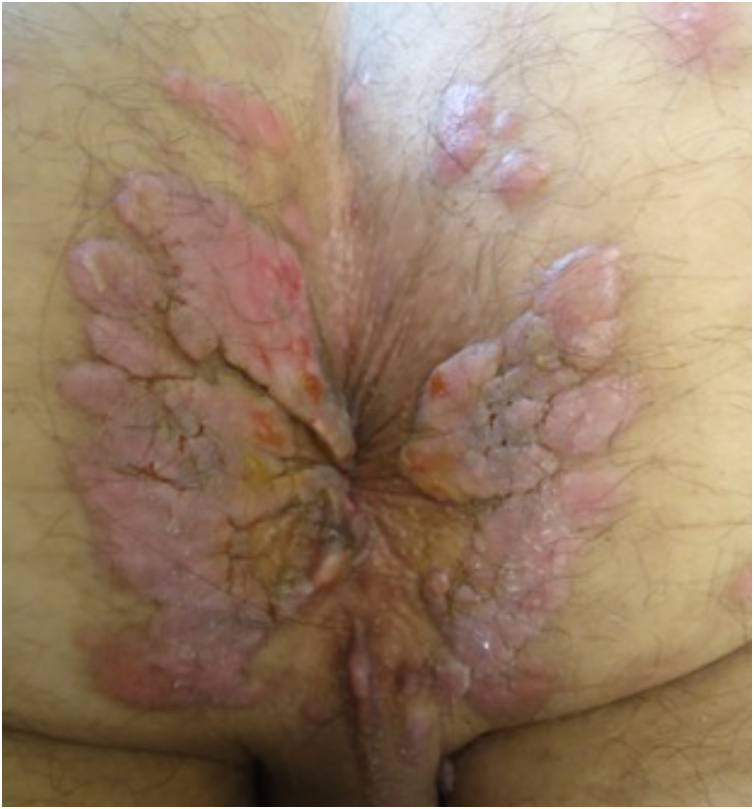
Perianal flat condyloma, clinical manifestation of secondary syphilis that is a differential diagnosis for condyloma acuminata.

**Fig. 10:**
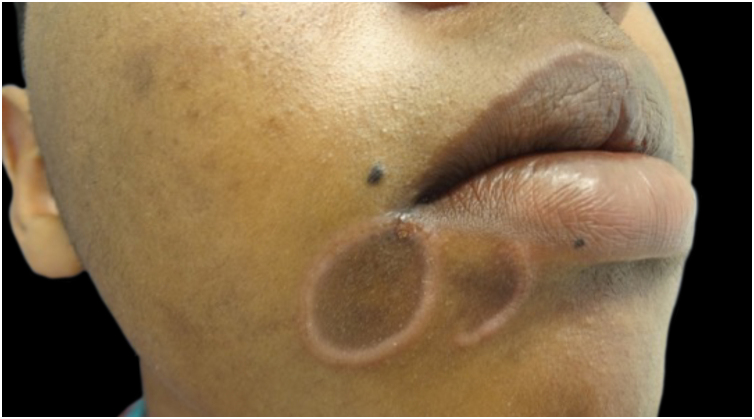
Perioral elegant syphilides, clinical manifestation of secondary syphilis in an Afro-descendant patient.

With persistence of the skin lesions, regression occurs in the affected body area, localized in segments of the skin, while the elementary lesions increase to nodules and plaques and may acquire a corymbiform aspect (Fig. 8).^32^, ^39^, ^40^ In skinfold areas, nodular and tumor lesions, called flat condylomas, appear (Fig. 9), which are extremely infectious and can be confused with condyloma acuminata caused by the human papillomavirus (HPV).^31^, ^34^, ^41^, ^42^ In melanodermic patients, facial lesions may acquire annular and circinate configurations, being called elegant or beautiful syphilides (Fig. 10).^26^

**Fig. 11:**
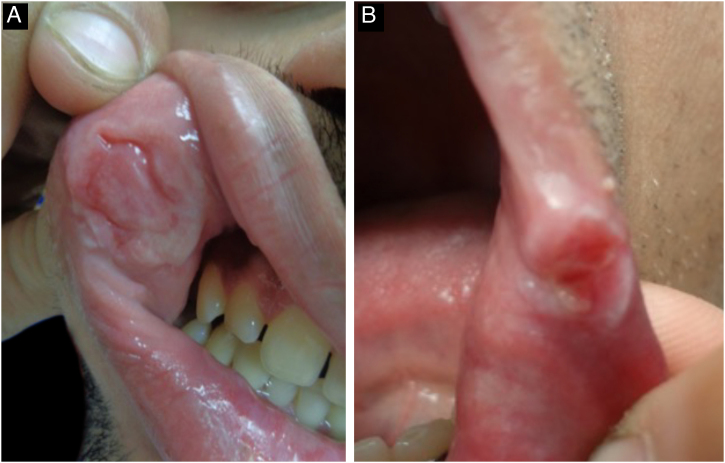
Oral mucosal lesions of secondary syphilis. A- linear erosions on the labial mucosa (“snail track ulcers”); B Papules with transverse erosions on the labial commissure (“split papules”).

Mucosal lesions are also common and characteristic of secondary syphilis, occurring in 30% to 40% of patients. Mucosal patches are exudative, oval, well-demarcated erosions with erythematous borders, most commonly manifesting on the tongue and lips; like flat condyloma, these lesions are highly infectious. Occasionally, erosions can coalesce and take on a linear outline, and are called “snail-track ulcers”. In the oral commissures, lesions may appear as papules with transverse erosions called “split papules” (Fig. 11).^22^, ^34^, ^43^, ^44^

**Fig. 12:**
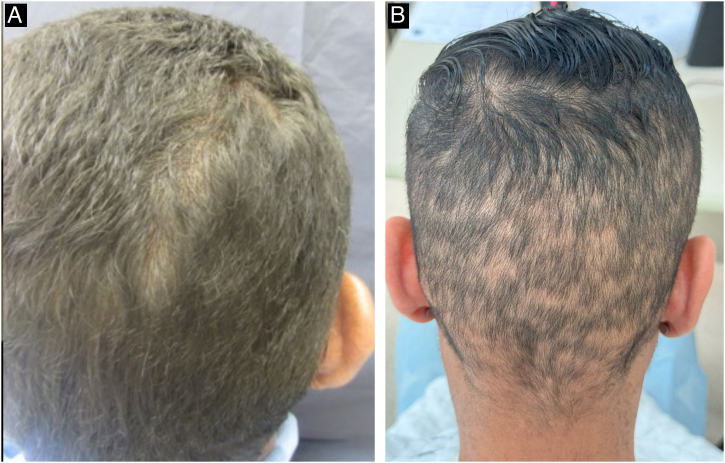
Alopecia in secondary syphilis. A - “moth-eaten” pattern; B - “clearing” pattern.

**Fig. 13:**
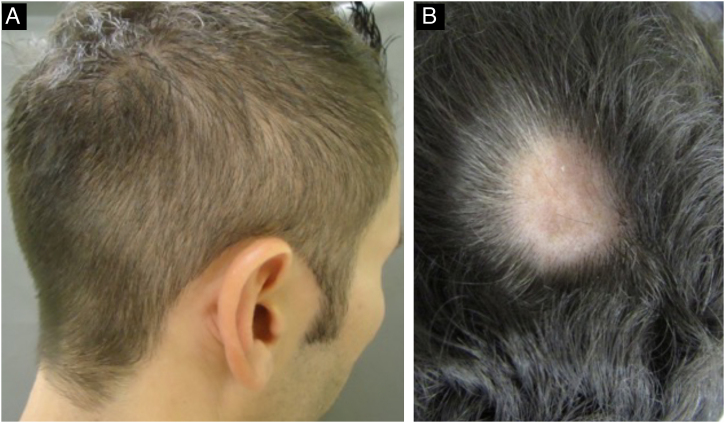
Manifestations of alopecia in secondary syphilis. A - Diffuse essential alopecia; B- Alopecia areata-like lesion.

During the secondary stage, in addition to the involvement of the skin and mucous membranes, changes in the skin appendages may also occur, i.e., hair and nails. Hair loss is also called syphilitic alopecia (SA) which is classified as symptomatic (when lesions occur on the scalp associated with hair loss) and essential, when only hair loss occurs. The latter is subdivided into three patterns: patchy alopecia, diffuse alopecia, and mixed alopecia. Essential patchy SA is the most common and is characterized by the presence of multiple patches of non-cicatricial alopecia, without inflammation or desquamation; it is also called “moth-eaten” or “in clearings” (Fig. 12). It occurs mainly in the parieto-occipital region, but can also appear on the beard, eyelashes, armpits, pubis, trunk and legs. Diffuse essential SA is caused by telogen effluvium-like hair loss (Fig. 13), while mixed essential SA is characterized by small irregular patches that develop together with diffuse alopecia.^45^, ^46^ In addition to these clinical forms, some authors describe a fourth pattern of essential SA: the alopecia areata-like type (Fig. 13).^22^

**Fig. 14:**
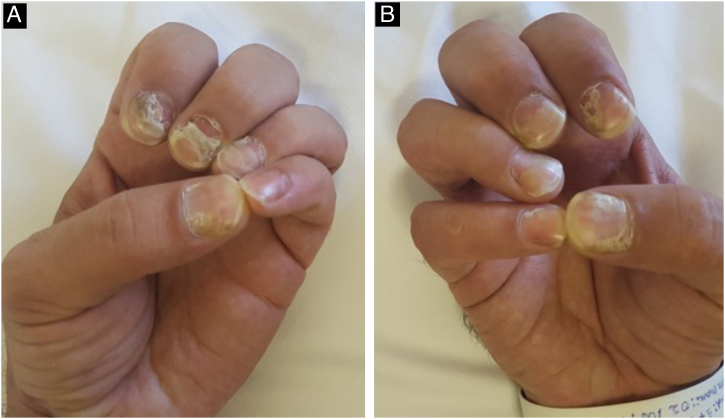
A and B- Involvement of the nail apparatus in a patient with secondary syphilis, characterized by onychomadesis.

Changes in the nail apparatus are rare. Changes in the nail plate result from the involvement of the matrix, causing fragility, splitting, fissures, corrosion, onycholysis, Beau's lines, onychomadesis (Fig. 14), and even loss of the nail. The onset of a periungual and/or subungual inflammatory leads to syphilitic paronychia, with erythema and edema in the periungual tissues.^22^, ^47^

Malignant syphilis, or nodular-ulcerative syphilis, is typically characterized by the presence of asymmetric ulcers or round necrotic plaques with lamellar or rupioid crusts located on the scalp, face, trunk, and extremities. Oral ulcers may occur with systemic signs and symptoms; fever, headache, and lymphadenopathy are usually present. It is more common in patients with HIV infection and low CD4 + T lymphocyte counts, malnourished patients, MSM, patients with previous syphilis, diabetes mellitus, tuberculosis, and alcohol abuse.^14^, ^18^, ^20^, ^22^

In addition to the skin manifestations caused directly by *Treponema pallidum* infection, there are reports of skin reactions secondary to the bacteria, considered reactive conditions, more common during secondary syphilis. The cases described are more common in patients with HIV coinfection and range from manifestations of Sweet's syndrome to erythema multiforme.^16^, ^48^, ^49^ They are diagnosed by associating the clinical picture with positive serology for syphilis and a reactive pattern on histopathology, with no evidence of the bacteria by immunohistochemistry. Unlike the Jarisch-Herxheimer condition, where exacerbation of skin lesions occurs after the institution of treatment, these reactive conditions appear before and improve after treatment.

Secondary lesions tend to regress spontaneously after four to 12 weeks. Most lesions do not leave scars, but anetoderma may occur, most commonly reported in patients with positive HIV serology.^16^, ^39^

### Neurosyphilis and ocular syphilis

Neurosyphilis results from treponemal invasion of the CNS, with an increasing number of cases described in immunocompetent and heterosexual patients.^13^ Invasion of the meninges by treponema occurs early, 12 to 18 months after infection, but disappears in 70% of the cases without treatment. When the infection persists, which occurs during any stage of the infection, neurosyphilis appears, which may be symptomatic or asymptomatic.^26^

In asymptomatic neurosyphilis, the patient does not have clinical manifestations, but there is evidence of CNS infection in the analysis of the cerebrospinal fluid (reactive VDRL, elevated protein or leukocyte count).^22^

In symptomatic neurosyphilis, the clinical picture is usually nonspecific and can develop at any time during the natural history of the disease. In patients with coinfection with the HIV virus, a more fulminant course usually occurs, while in immunocompetent individuals, the disease is more insidious, with nonspecific symptoms.^13^, ^22^

The most common first symptoms of neurosyphilis are mild meningeal signs, such as headache and nausea. Cranial nerve palsies may occur, with unilateral or bilateral hearing loss, with or without tinnitus and nystagmus. Meningitis can cause fever, meningismus, and photophobia. In meningovascular syphilis, arteritis causes infarctions in the brain or spinal cord. The investigation of recent neurosyphilis should be performed in syphilitic patients with neurological or ocular signs, immunosuppression, or those who do not reduce VDRL levels after treatment.

Late symptomatic neurosyphilis, rare in the antibiotic era, most commonly causes general paresis (also called general paresis of the insane or paralytic dementia), which can manifest with dementia, seizures, and other psychiatric manifestations. Tabes dorsalis may also occur, which can manifest as fulminant pain, urinary incontinence, and erectile dysfunction, ataxia, Argyll-Robinson pupil (reacting to accommodation/focusing, but not to light), loss of reflexes, and impaired vibratory sensation.^22^ In a review of 137 articles, reporting on 286 patients with neurosyphilis, only 10% had coinfection with HIV. The most relevant clinical presentations were general paresis (49% of cases), manifested by cognitive impairment and psychiatric changes, followed by syphilitic meningitis (22%), meningovascular syphilis (11.5%), tabes dorsalis (11.5%), parenchymal gummas (3.5%) and epilepsy (2%).^13^

Ocular syphilis is considered a type of neurosyphilis. While most cases of syphilitic meningitis are accompanied by ocular involvement, ocular syphilis is not always accompanied by syphilitic meningitis.^13^, ^50^ Therefore, ocular syphilis should be suspected in any case of unexplained ocular inflammation. The disease can occur up to six weeks after transmission and be the only presenting feature of systemic syphilis. The most common findings are panuveitis and posterior uveitis, but ocular involvement can manifest in a variety of ways, affecting both the anterior segment of the eye (conjunctiva, cornea, and sclera) and the posterior segment (choroid and retina). These manifestations rarely occur in the primary stage, except as hard chancres located on the eyelid and conjunctiva. Keratitis, iris nodules, iridocyclitis, episcleritis, and scleritis may occur early in secondary syphilis, and chorioretinitis and vitritis may occur later in the secondary stage. However, ocular involvement is even more frequent in the late, latent, and tertiary stages of syphilis.^50^, ^51^

### Latent syphilis

Latent syphilis occurs when serological tests are positive but there is no clinical evidence of infection. The period of up to one year after contamination is classified as early latent syphilis, and from this date onwards, late latent syphilis begins.^51^

The diagnosis is generally based on situations in which tests are requested for patients without clinical symptoms, such as sexual partners or contacts of patients diagnosed with syphilis, routine or screening tests.

Based on studies that monitored the natural progression of syphilis, one-third of patients who had regression of secondary lesions achieve clinical and serological cure, one-third will develop no symptoms but will maintain positive non-treponemal serological tests, and the last third will have the disease progress to tertiary syphilis years to decades after the infection.^52^, ^53^

### Tertiary syphilis

Tertiary syphilis is rare and may manifest with mucocutaneous, cardiac, ophthalmological, neurological, skeletal, or gastric alterations.^54^, ^55^, ^56^, ^57^ The incidence of this phase of the disease decreased drastically with the use of penicillin in the treatment of the initial phases.^58^

The skin is the most affected organ. Cutaneous tertiary syphilis is classified as nodular and gummatous; the former shows dermo-epidermal involvement and the latter, hypodermal involvement.^54^, ^57^ The lesions of the nodular form are generally asymmetrical, chronic in appearance, painless, and slowly progressive in growth. The nodules are usually located on the face, interscapular areas, and extremities. These lesions may remain isolated, coalesce to form plaques or tumors, be distributed in an arciform pattern, or ulcerate.^54^, ^58^, ^59^, ^60^, ^61^ The gummatous form presents as firm and painless subcutaneous nodules, usually solitary, which later develop ulcerations and drain solid necrotic material. These are destructive lesions that can invade deep into the tissue and bone, healing with deeply retracted scars. Due to the frequent involvement of the cardiovascular system, especially the ascending aorta, adequate cardiac investigation is recommended.^13^, ^22^, ^54^, ^58^^,^^62^, ^63^

### Syphilis in patients living with HIV

It is known that syphilis can increase the risk of acquiring and transmitting HIV by two to ninefold, mainly due to genital ulcers; while HIV infection and antiretroviral therapy (ART) can, in additionally facilitate infection due to a decrease in the immune response mediated by T cells, and alter the natural history and clinical presentation of syphilis.^64^, ^65^

In patients living with HIV, syphilis can present concomitant clinical manifestations of two different clinical stages, in addition to a greater predisposition to the formation of atypical, larger, and deeper lesions. Multiple primary chancres, a greater number of ulcerated lesions, and early malignant syphilis in the secondary form, systemic manifestations such as uveitis, aortitis, encephalitis, arthritis, gastric and hepatic involvement can be observed. The rate of neurological involvement is high, and this involvement is often early and asymptomatic.^66^, ^67^, ^68^

## Diagnosis

The diagnosis of syphilis is clinical, laboratory based and can be confirmed by several methods, such as direct treponema detection and serological reactions (immunological tests), with the latter being the most commonly used. Direct treponema detection can be performed by dark-field microscopy (sensitivity of 74% to 86%), direct immunofluorescence, rapid tests, examination of stained material, and histopathological examination of tissue biopsies. These tests are called direct tests and are extremely important for confirmation of the diagnosis of syphilis.

Direct tests can be performed in symptomatic cases of syphilis, but in most cases, they are performed to investigate conditions with nonspecific skin lesions. Dark-field treponema testing, stained material examination, and histopathological examination of tissue biopsies are important for confirming the diagnosis of symptomatic phases of the disease. However, these tests are not always available in most health services. Immunological tests can be used in both the symptomatic and asymptomatic (latency) phases. The most commonly used immunological tests in clinical practice are treponemal and non-treponemal tests.

According to the Clinical Protocol and Therapeutic Guidelines for Comprehensive Care for People with Sexually Transmitted Infections (STIs) of the Brazilian Ministry of Health, asymptomatic individuals with a reactive non-treponemal test of any titer and a reactive treponemal test, with no record of previous treatment, should be considered as having acquired syphilis. This is an operational recommendation, since false-reactive tests can occur, and antimicrobial treatments for other diseases can also cure syphilis, in addition to the fact that there may be spontaneous resolution of untreated treponematosis. Likewise, individuals symptomatic for syphilis, with at least one reactive test, treponemal or not, are also considered cases of acquired syphilis.^19^

Whenever possible, it is recommended to start disease investigation with a treponemal test, preferably the rapid test (RT), and then associate a non-treponemal test to increase the positive predictive value of the initial test. It should be noted that rapid tests are not available in all health centers in the country.

### Immunological tests

#### Treponemal tests

Treponemal tests detect specific antibodies produced during the initial immune response against *T. pallidum* antigens. Therefore, they are the first to test positive and remain positive, in most cases, for the rest of the patient life, even after specific treatment. Individuals who have already been treated but who have a clinical epidemiological picture suggestive of syphilis should undergo a non-treponemal test for possible new treatment.

Hemagglutination and passive hemagglutination tests (PHAT), indirect immunofluorescence test (Fluorescent Treponemal Antibody – Absorption test ‒ FTA-Abs), chemiluminescence, indirect immunoenzymatic assay, and rapid tests are treponemal tests. RT mainly uses the lateral flow immunochromatography or dual path platform (DPP) methodology and, according to the Brazilian Ministry of Health, is available in all health units of the Unified Health System (SUS, *Sistema Único de Saúde*).

#### Non-treponemal tests

Non-treponemal tests become positive after the host immune recognition of the treponema (action of anti-treponemal antibodies) when the bacteria degrade and release cardiolipin components from its cell structure. Thus, these tests detect non-specific anticardiolipin antibodies for *T. pallidum* antigens and are important both for diagnosis and for monitoring treatment response. The Venereal Disease Research Laboratory (VDRL), RPR (Rapid Test Reagin), and TRUST (Toluidine Red Unheated Serum Test) are examples of these tests.

Whenever a non-treponemal test is performed, it is important that the pure and diluted sample are employed, due to the prozone phenomenon.^69^ In the case of test reactivity, the sample should be diluted, using a dilution factor of two, until the last dilution at which there is no more reactivity in the test. The final result of the reactive tests should be expressed in titers (1:2, 1:4, 1:8, etc.). Non-treponemal tests, although nonspecific, can be used in diagnosis (as a first test or complementary test) and also to monitor the response to treatment and control of cure. An adequate drop in titers is an indication of treatment success.

VDRL and RPR are useful and inexpensive tests, but nonspecific; false-reactive results, although rare, can occur. Anticardiolipin antibodies may be present in lepromatous leprosy, Lyme disease, HTLV-1, malaria, tuberculosis, and other diseases in which lysis of cells containing cardiolipin in their structure occurs, such as occurs in several pathogens and the human cell itself (Table 1)^70^. In patients with a clinical picture suspicious for syphilis and nonspecific tests with low titers, treponemal tests with high specificity, such as RT, FTA-Abs, PHAT, MHA-TP or others, should be performed. If none of these tests are not available, it is advisable that the patient be treated as having syphilis.

**Table 1:** Situations that can generate false-reactive results in non-treponemal tests.

Situations that may generate transient false-reactive results	Situations that can generate permanent false-reactive results
After immunizations	Injectable drug use
After myocardial infarction	Autoimmune diseases (antiphospholipid antibody syndrome and systemic lupus erythematosus, others)
Some febrile infectious diseases (malaria, hepatitis, varicella, measles, infectious mononucleosis, others)	HIV infection
Pregnancy	Leprosy
	Chronic hepatitis
	Advanced age

The isolated analysis of a single non-treponemal test result can lead to diagnostic errors and inappropriate therapeutic decisions. High titers in adequately treated patients may be gradually decreasing and low titers can occur in three situations: recent infection, late stages of infection (late syphilis), individuals adequately treated but who have not yet tested negative or won’t do so (serological scarring). The term serological scarring is used in situations in which an individual, proven to have been treated, shows a decrease in titer in two dilutions, but still shows reactivity in the tests. In these cases, treponemal tests tend to be reactive, and quantitative non-treponemal tests will show low titers (≤ 1:4).

Overall, immunological tests for syphilis in PLHIV do not show any changes when compared to those performed in non-coinfected individuals. However, in PLHIV, there may be a higher frequency of high dilutions, a longer time for tests to become negative, as well as false-negative results.

In patients not co-infected with HIV, the false-negative rate varies from 1% to 2%, while in co-infected patients, it can reach 10%. This may occur due to patients inability to develop an immune response with the production of antibodies against *T. pallidum*. Regarding non-treponemal tests (VDRL), there is also an increase in false negative results related to the prozone effect. This effect may be related to the anomalous function of B cells, leading to an increase in the production of antibodies against various antigens.^71^

According to the Clinical Protocol and Therapeutic Guidelines for the Management of HIV Infection in Adults, from the Ministry of Health, in the clinical follow-up of PLHIV, an immunological test should be performed every six months or after any risky sexual exposure.^72^

### Dark-field treponema testing

Dark-field treponema testing is performed using samples obtained from primary or secondary syphilis lesions in adults or children. Collecting material from lesions in the oral cavity is not recommended due to the presence of other saprophytic spirochetes, which may result in false reactive tests. The test should be performed on fresh serous exudate from the lesion, avoiding erythrocytes, other organisms, and tissue debris. The collected sample should be immediately analysed on a microscope with a dark-field condenser to look for *T. pallidum*.

To identify *T. pallidum* using this technique, it is important to observe its morphology, size and typical movements. The spirochete consists of a thin organism (0.10 to 0.18 μm wide), 6 to 20 μm long, and with 8 to 14 regular spirals. *T. pallidum* moves quickly, and it is possible to identify elongation and shortening movements; it rotates relatively slowly around its longitudinal axis, in addition to performing syncopated flexions and twists in its central region. In dark-field microscopy, the spirochetes appear as bright, white spiral bodies, illuminated against a black background.^70^

### Examination of stained material

Sample collection for this examination should be performed in the same manner as is done for direct examination of fresh material. The available methods are:

• Fontana-Tribondeau method: the sample is smeared and allowed to dry on the slide and then stained with silver nitrate, which will impregnate the cell wall of the treponema, thus allowing the parasite to be seen under the microscope;
• Burri method: this technique is performed with Indian ink;
• Giemsa staining method: *T. pallidum* is stained faintly (palely), making it difficult to observe the spirochetes;
• Levaduti method: uses silver in histological sections.

The sensitivity of the examination of stained material is lower than that of dark-field treponema testing.^70^

### CSF puncture

CSF puncture is indicated in cases of suspected neurosyphilis and is recommended in specific situations for syphilis diagnosis and patient follow-up after treatment has been implemented. It is worth emphasizing that the CSF analysis should include cytology, biochemical profile, and VDRL in the material.

Lumbar puncture is indicated for neurosyphilis screening in the following cases: presence of neurological or ophthalmological symptoms; in case of evidence of active tertiary syphilis and after clinical treatment failure without sexual re-exposure. For PLHIV, lumbar puncture is indicated after treatment failure, regardless of sexual history.

In the follow-up of a patient initially treated without neurosyphilis, the procedure is recommended when there is suspected treatment failure, that is, failure of the non-treponemal test to decrease in outpatient serological follow-up. The puncture is also indicated for patients diagnosed with neurosyphilis; the test should be performed every six months for laboratory monitoring of their infection. Treated patients who do not show the expected reduction in VDRL titers should be investigated through cerebrospinal fluid puncture for the possibility of neurosyphilis.

Although it is not considered a criterion by the Ministry of Health, some authors indicate the test for all patients coinfected with syphilis and HIV, regardless of the clinical stage, who meet at least one of the following criteria:^72^

• Neurological or ophthalmological signs or symptoms;
• Evidence of active tertiary syphilis (aortitis, syphilitic gummas, among others);
• After clinical treatment failure.

## Treatment

Penicillin has been the mainstay of syphilis treatment since it became widely available in the late 1940s. *T. pallidum* resistance to penicillin has never been reported, and since this bacterium divides more slowly than others, it is necessary to maintain penicillin levels in the blood above the minimum inhibitory concentration for at least ten days, which is achieved by administering a single intramuscular injection of long-acting benzathine penicillin G.

### Primary, secondary and recent latent syphilis

The first-line antibiotic is benzathine penicillin G, at a total dose of 2,400,000 IU, administered intramuscularly, in a single dose – 1,200,000 IU is administered in each buttock. Doxycycline, 100 mg, orally, twice a day for 15 days, can be used as an alternative drug (except for pregnant women).

### Tertiary and late latent syphilis

Benzathine penicillin G is administered at a dose of 2,400,000 IU intramuscularly once a week for three weeks, totaling 7,200,000 IU. The alternative drug is doxycycline, 100 mg, orally, twice a day for 30 days (except for pregnant women). For pregnant women proven to be allergic to penicillin, desensitization is recommended in a tertiary service, according to existing protocols.

Individuals with a confirmed diagnosis of syphilis, whose disease duration cannot be determined, should be treated as having late latent syphilis.

### Neurosyphilis and ocular syphilis

Since penicillin G benzathine is not able to cross the blood-brain barrier, treatment of neurosyphilis is hospital-based, carried out with crystalline penicillin, 18-24 million units per day, intravenously, administered in doses of 3-4 million IU, every four hours, for 14 days. Ceftriaxone, 2 g, administered intravenously, once a day, can be used as an alternative drug, for ten to 14 days.

### Children

Recent phase: penicillin G benzathine, 50,000 IU/kg, intramuscularly, in a single dose.

Late phase: 50,000 IU/kg of body weight, intramuscularly, once a week, for three weeks.^73^

According to the Brazilian Ministry of Health, given the current epidemiological scenario, immediate treatment with benzathine benzylpenicillin is recommended, after only one reactive test for syphilis (treponemal or non-treponemal test) for the following situations (regardless of the presence of signs and symptoms of syphilis): pregnant women, victims of sexual violence, individuals with a chance of loss to follow-up, individuals with signs and/or symptoms of primary or secondary syphilis and individuals without a previous diagnosis of syphilis.

### People living with HIV

In PLHIV, treatment should be carried out in a similar way to that of non-co-infected individuals. To date, in Brazil, there is no evidence of *T. pallidum* resistance to benzathine penicillin.

In PLHIV with early syphilis, there may be an increased risk of neurological complications and higher rates of inadequate serological response following the use of recommended regimens. Although data are limited, no syphilis treatment regimen has been shown to be more effective in preventing neurosyphilis in PLHIV than the syphilis regimens recommended for the general population.^74^

Careful follow-up after therapy is essential, and the use of antiretroviral therapy according to current HIV guidelines may improve clinical outcomes among people co-infected with HIV and syphilis. Differences in treatment response in HIV-syphilis co-infected patients may not apply to those with virological suppression of HIV.^74^

### Jarisch-Herxheimer reaction

The Jarisch-Herxheimer reaction is characterized by the exacerbation of pre-existing skin lesions, associated with pain or pruritus, general malaise, fever, chills, headache, and arthralgia. This clinical picture is due to the massive destruction of treponemes by treatment, which causes an antigen storm in the host that induces an inflammatory response in the same proportions. Thus, it is more common in the treatment of secondary forms, mainly those with high titers in non-treponemal tests, and can occur during the 24 hours after the first dose of penicillin. This picture usually regresses spontaneously in 24 to 48 hours. If necessary, analgesics can be used.^75^

The main differential diagnosis is allergy to benzathine penicillin. However, this condition is rare and is usually characterized by the presence of urticarial lesions.

### Monitoring after treatment

Non-treponemal tests are important in monitoring the cure of patients and should be performed three, six and 12 months after treatment.

An adequate immunological response to treatment is accepted when there is a decrease in the titer of two dilutions of non-treponemal tests within six months in individuals with recent syphilis, and two dilutions within twelve months in cases of late syphilis.^76^ Seroreversion (non-reactive non-treponemal test) or progression to serological scarring may occur. Negative treponemal tests are not expected after the patient is cured.

Patients, including people living with HIV, should be monitored after completing treatment for syphilis using a non-treponemal test, preferably the same diagnostic method, every three months until the 12th month of patient follow-up, and every six months until the 24th month for patients whose tests were not negative. Pregnant women should be tested monthly.

### Control actions

One of the pillars of the control actions for acquired syphilis is the continuous encouragement of educational activities for all STIs, encouraging their prevention through the use of condoms. Early diagnosis of the disease should be encouraged by expanding the supply of rapid tests in the public health system. It is also important that health professionals, especially those working in primary care, receive continous training for early diagnosis, correct treatment, adequate follow-up, and reporting of syphilis cases.^77^

Evaluation and treatment of sexual partners are crucial for interrupting the chain of syphilis transmission. For partners that report exposure to a person with syphilis, within 90 days, it is recommended that these sexual partners be offered presumptive treatment (regardless of the clinical stage or signs and symptoms), with a single dose of benzathine benzylpenicillin 2.4 million IU, IM (1.2 million IU in each buttock).

In addition to promoting safe sex based on condom use, other prevention measures are important and complementary to safe sexual practices, such as immunization for hepatitis B, C, and HPV; knowledge of the HIV serological status of the sexual partner(s); regular testing for HIV and other STIs; testing of all PLHIV; performing preventive exams for cervical cancer; performing pre-exposure prophylaxis (when indicated); access to contraception and conception and performing post-exposure prophylaxis (when indicated).^70^

It should be noted that, in the case of suspected syphilis or when unprotected sexual contact is referred, testing should be indicated, in endemic areas, not only for treponematosis but also for other detectable STIs such as HIV, hepatitis and HTLV.

## Financial support

None declared.

## Authors' contributions

Carolina Talhari: Design and planning of the study; collection of data, or analysis and interpretation of data; drafting and editing of the manuscript or critical review of important intellectual content; collection, analysis and interpretation of data; effective participation in research orientation; intellectual participation in the propaedeutic and/or therapeutic conduct of the studied cases; critical review of the literature; approval of the final version of the manuscript.

Kaique Arriel: Drafting and editing of the manuscript or critical review of important intellectual content; collection, analysis and interpretation of data; critical review of the literature.

Marcio Soares Serra: Approval of the final version of the manuscript; drafting and editing of the manuscript or critical review of important intellectual content.

John Verrinder Veasey: Design and planning of the study; collection of data, or analysis and interpretation of data; drafting and editing of the manuscript or critical review of important intellectual content; collection, analysis and interpretation of data; effective participation in research orientation; intellectual participation in the propaedeutic and/or therapeutic conduct of the studied cases; critical review of the literature; approval of the final version of the manuscript.

## Conflicts of interest

None declared.
